# The Development of Nanoalumina-Based Cement Mortars for Overlay Applications in Concrete Floors

**DOI:** 10.3390/ma12213465

**Published:** 2019-10-23

**Authors:** Jacek Szymanowski, Łukasz Sadowski

**Affiliations:** Faculty of Civil Engineering, Wroclaw University of Science and Technology, Wybrzeże Wyspiańskiego 27, 50-370 Wrocław, Poland; lukasz.sadowski@pwr.edu.pl

**Keywords:** aluminum oxide nanopowder, cement mortar overlay, substrate, concrete floors, adhesion, functional properties

## Abstract

This article focuses on the development of nanoalumina-based cement mortars for overlay applications in concrete floors. It focuses on the effect of applying aluminum oxide (Al_2_O_3_) nanopowder to the cement mortar used to make the overlay, on the adhesion of this overlay to concrete substrate and on its functional properties. It was claimed that the addition of 0.5% of Al_2_O_3_ nanopowder has a positive effect on the adhesion of the cement mortar used to make the overlay to the substrate made of concrete. The prior studies performed using scanning electron microscopy (SEM) confirmed that the reason for the improvement in adhesion is the fact that cement mortar used to make the overlay with the addition of 0.5% of Al_2_O_3_ nanopowder is less porous than the reference mortar within the interphase. The article concurs that the most favorable results, in terms of lower abrasion resistance and higher subsurface tensile strength of the cement mortar used to make the overlay, are mainly brought about by adding 0.5% of Al_2_O_3_ nanopowder.

## 1. Introduction

In construction, in terms of durability, cement mortar used to make the overlay should primarily have an adequate adhesion to the concrete substrate [1,2,3]. According to [4,5,6,7], the pull-off adhesion of cement mortar used to make the overlay to the concrete substrate should be at least 0.5 MPa for newly made overlays. As highlighted in [8], in the case of repaired concrete elements, the value of pull-off adhesions should be at least 2 MPa and 1 MPa for structural and nonstructural repairs, respectively. As stated in [9,10,11,12], the properties of the cement mortar used to make the overlay are strongly influenced by the porosity, microcracks, moisture content, absorption rate and morphology of the substrate. Thus, according to recent literature, in order to obtain this adhesion at an appropriate level, mechanical treatment of the concrete substrate surface is applied [13,14,15]. Although sandblasting seems to be the most advantageous for many surfaces [16], as demonstrated in [17] for layered cement composites, it is beneficial to use shot-blasting in order to most efficiently treat the concrete substrate. Furthermore, texturing the surface of a concrete substrate has recently gained more attention [18,19]. What is more, surface exposure of the coarse aggregate of the substrate, and strengthening of the surface of the concrete substrate, are frequently done [20,21,22]. Increasingly, various additives are also being used to modify the material of the cement mortar used to make the overlay [23,24,25,26,27,28,29,30,31,32]. For example, Luković et al. [33] recently used blast furnace slag to replace part of the Portland cement in repair overlay.

Moreover, for durability reasons, the cement mortar used to make the overlay should have suitable mechanical and functional properties. The mechanical properties include mainly the compressive and flexural strength. On the other hand, the functional properties include mainly subsurface tensile strength, abrasion resistance and hardness. Thus, additional treatments should be applied in in order to obtain the values of mechanical and functional properties at the desired levels. The literature also presents the use of polypropylene, steel, copper and basalt fibers in mortars and concretes to improve their abrasion resistance [34,35,36,37].

It seems that the material modification of the composition of the cement mortar used to make the overlay with mineral additives, with particular emphasis on nanoparticles, is reasonable [38,39,40,41,42]. The application of nanoparticles improves some of the properties of cement-based mortars, such as the corrosion protection of reinforcing steel [43] and the mitigation of the alkali-silica reaction [44]. There is hope that this approach can also successfully improve the adhesion of the cement mortar used to make the overlay to the concrete substrate, as well as improve its functional properties. Based on an analysis of the subject of the literature concerning, e.g., the application of nanoparticles as an addition to the cement mortar used to make the overlay [45,46,47,48,49,50,51,52,53], one can see opportunities to improve its adhesion with the substrate and improve its functional properties.

The results of research carried out by the authors of work [54] indicate that the modification of the composition of the cement mortar used to make the overlay by adding amorphous silicon oxide (SiO_2_) nanospheres slightly increases its adhesion with the concrete substrate, and also significantly improves its functional properties. However, according to the authors of work [54], further research should primarily focus on the search for an additive that will improve the adhesion of the cement mortar used to make the overlay to the substrate more than the addition of SiO_2_ nanoparticles in the form of amorphous nanospheres. According to the authors, it is worth conducting research on the impact of the content of nanoparticles not used for this purpose; e.g., aluminum oxide (Al_2_O_3_). Stefaniuk et al. [55] successfully evaluated the elastic properties of self-compacting concrete with Al_2_O_3_ nanoparticles. Chen et al. [56] studied the early hydration of calcium aluminate cement modified using Al_2_O_3_ nanoparticles. In regard to layered composites, the recently performed applications of adding Al_2_O_3_ nanoparticles have had a positive effect and allowed for an increase in the adhesion of epoxy resin to steel [57], had a positive effect on the adhesion of the geopolymer overlay to the concrete substrate [58], improved the adhesion of concrete to reinforcing steel [59] and had a positive effect on improving the strength parameters of epoxy adhesives in aluminum joints [60].

Considering the above, there has not yet been broader research on the effect of the modification of the material of cement mortar used to make the overlay using Al_2_O_3_ nanopowder on its adhesion with the concrete substrate. Moreover, the impact of the content of Al_2_O_3_ nanopowder is still unknown. To date, the effects of this nanopowder on the functional properties of the cement mortar used to make the overlay have not yet been studied. Therefore, the purpose of this article is to obtain the answer to the above questions. Hopefully, getting the answers for the mentioned questions will lead to the development of nanoalumina-based cement mortars for overlay applications in concrete floors.

## 2. Materials and Methods

### 2.1. Concrete Substrate Mix Design and Preparation

**Figure 1:**
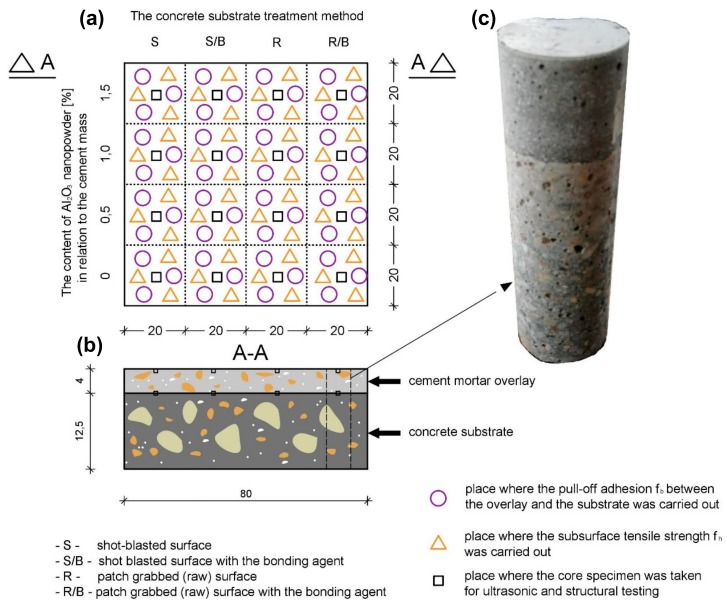
Model element made of cement-based composites: (**a**) view from the top, (**b**) cross-section and (**c**) view of the core specimen for structural testing (description in the text).

The tests were carried out on a model element with dimensions of 800 × 800 mm^2^. This element was made of cement-based composites: an overlay made of cement mortar and a substrate made of concrete. The total thickness of this element was equal to 165 mm (Figure 1). The overlay of the element had a thickness of 40 mm. The thickness of the substrate was equal to 125 mm.

The substrate was made of concrete, with a water-cement ratio equal to 0.47. The following materials were used to make the substrate (per 1 m^3^): 352.0 kg of Portland cement type CEM II A-LL 42.5 R (Cement Hranice, Italian Buzzi Unicem group, Hranice, Czech Republic); 165 kg of water; 40 kg of fly ash (Zespół Elektrociepłowni Wrocławskich “Kogeneracja S.A”, Wrocław, Poland); 724.4 kg of fine aggregate with a bulk density of 2.62 g/cm^3^ (Mineral mine WIKA, Paniowice, Poland); and 1086.6 kg of coarse aggregate with a maximum size of grain equal to 8 mm and a bulk density of 2.60 g/cm^3^ (Mineral mine “Byczeń”, Byczeń, Poland). In order to obtain a consistency class S3 (slump from 100–150 mm) of the concrete mix, 2.0 L/m^3^ of polycarboxylate-based plasticizer was used (Sika, Wroclaw, Poland). The density of this plasticizer was equal to 1.07 g/cm^3^. The water-cement ratio was 0.5. The maturation conditions of the substrate were the ambient temperature equal to 20 ± 3 °C and the humidity equal to 60% ± 5%. This concrete composition is commonly used to make substrates in layered elements in civil engineering (such as for example floors). After that, the surface of the concrete substrate was divided into four parts (Figure 1). Each of the parts was treated in different ways, which allowed four types of surfaces with different morphology to be obtained:

1. S—shot-blasted surface obtained after shot-blasting with removal of dust;
2. S/B—shot-blasted surface obtained after shot-blasting with removal of dust and the application of the bonding agent;
3. R—patch grabbed (raw) surface obtained after casting;
4. R/B—patch grabbed (raw) surface obtained after casting and the application of the bonding agent.

As the bonding agent, the ready-made mix based on synthetic resin (Weber PRIMO, Saint—Gobain Construction, Polska sp. z o.o., Warsaw, Poland) was applied. The bonding agent was applied on the surface of the substrate 4 h before casting the material of cement mortar used to make the overlay.

### 2.2. Mix Design of the Cement Mortar Used to Make the Overlay and Its Preparation

In this research, Al_2_O_3_ nanopowder (Sigma Aldrich, Poznan, Poland); Portland cement type CEM I 42.5 R with a bulk density of 1.106 g/cm^3^ containing 64.07% CaO, 19.98% SiO_2_, 4.95% Al_2_O_3_, 2.66% Fe_2_O_3_, 1.45% MgO, 0.73% K_2_O and 0.18% Na_2_O (Cementownia Górażdże Cement S.A. Heidelberg Cement Group, Górażdże, Poland); fine aggregate (sand) with a bulk density of 1.497 g/cm^3^ (mineral mine “Margo”, Mietkow, Poland); and polycarboxylate-based superplasticizer with a density of 1.080 g/cm³ (Sika, Wroclaw, Poland) were used to make the cement mortar in the proportions given in Table 1. The water-binder ratio for this mortar was equal to 0.3. The mixing procedure was as follows. The superplasticizer was added to the mixing. Then, Al_2_O_3_ nanopowder was added to the water and mix. Next the cement was added and mixed for 45 s using the rotation speed equal to 140 rpm (automatic mixer was used). Then sand was added and all was mixed for another 45 s using the same rotation speed. After that, the mix was mixed again for 18 s using the rotation speed equal to 285 rpm. After casting the cement mortar used to make the overlay, the maturation conditions were 21 ± 1 °C and 60% ± 5% humidity.

### 2.3. Determination of the Particle Size Distribution of Nanopowder Using Transmission Electron Microscopy (TEM)

The morphology of the Al_2_O_3_ nanopowder was examined with transmission electron microscopy (TEM) imaging using a Hitachi H-800 electron microscope (Hitachi, Tokyo, Japan). The powder was suspended in deionized water and macroscopic aggregates were ultrasonically partitioned for 1 s. The solution was immediately put on the standard carbon-on-copper supporting grids with a volume of 4 µL, drained of most liquid with filtering paper, then air dried for 1 h. Observations were made in standard bright-field mode, using an accelerating voltage of 150 kV and an EMSIS Quemesa CCD camera (EMSIS GmbH, Muenster, Germany).

### 2.4. Determination of the Consistency and Bulk Density of Fresh Mortars

Before laying the cement mortars used to make the overlay, their consistency was determined using a Novikow cone (MERAZET S. A., Poznań, Poland) according to [61]. The fresh mortar was placed in the measuring vessel and the metal cone was placed over the mortar surface (the vertex of the cone touched the surface) Then the metal cone was falling vertically for 10 s. After this time, the measurements were taken from the scale on the side surface of the cone. The measurement was repeated three times. In order to determine the bulk density of the fresh mortar according to standard [62], the fresh mortar was placed in the mold with specific volume and mechanically compacted and weighted. Then the bulk density was calculated. The measure of bulk density was taken three times. The setting times of fresh mortar were carried out using Vicat apparatus (MERAZET S. A., Poznań, Poland) according to standard [63].

### 2.5. Determination of the Mechanical Properties and Porosity of Hardened Cement Mortars

According to [64], 6 samples with size of 40 × 40 × 160 mm^3^ were concreted from each mix in order to determine the mechanical properties and porosity of hardened cement mortars. Among others, the total porosity *p*, the compressive strength *f*_c_ and flexural strength *f*_ct_ were determined. The samples were stored at 21 ± 1 °C and humidity up to 90% and tested after 28 days. First, flexural strength tests were carried out on six samples with dimensions 40 × 40 × 160 mm^3^. Then 12 halves of these samples were used for compressive strength tests (6 samples with dimensions 40 × 40 × 80 mm^3^) and to determine the total porosity of hardened mortars (6 samples with dimensions 40 × 40 × 80 mm^3^). The total porosities *p*, were determined using a Le Chatelier volume vessel according to Equation (1):

(1) $$p=\frac{\rho -\rho_{0}}{\rho }100$$

ρ—density (kg/cm^3^), $\rho_{0}$—bulk density (kg/cm^3^).

### 2.6. Determination of the Pull-Off Adhesion of the Cement Mortars Used to Make the Overlays to the Concrete Substrate

**Figure 2:**
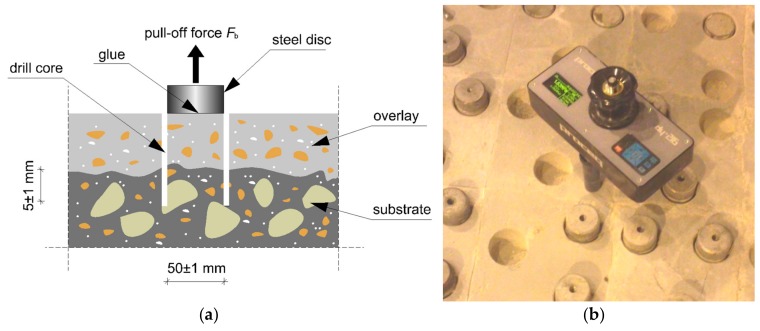
The pull-off method: (**a**) the scheme and (**b**) the view of test stand.

After 28 days, pull-off adhesion *f*_b_ tests were carried out using the pull-off method according to [65]. On Figure 2 the scheme of this method has been presented. The procedure is as follows: the drill of the core in the cement mortar is used to make the overlay with a diameter *D*_f_ = 50 mm and 5 mm. below overlay depth is performed, then the steel disc is glued to the overlay. Next, the steel disc is pulled off the substrate together with pulling off strength measuring. The loading rate should be equal to 0.05 MPa/s. The pull-off adhesion *f*_b_ between the cement mortar used to make the overlay and the concrete substrate was calculated according to Equation (2):

(2) $$f_{b}=\frac{4F_{b}}{\pi D_{f}^{2}}$$

$F_{b}$—failure force (N), $D_{f}^{}$—the diameter of the core (m).

### 2.7. Determination of the Course of the Longitudinal Velocity of the Ultrasonic Wave along the Thickness of the Cement Mortar Used to Make the Overlay

**Figure 3:**
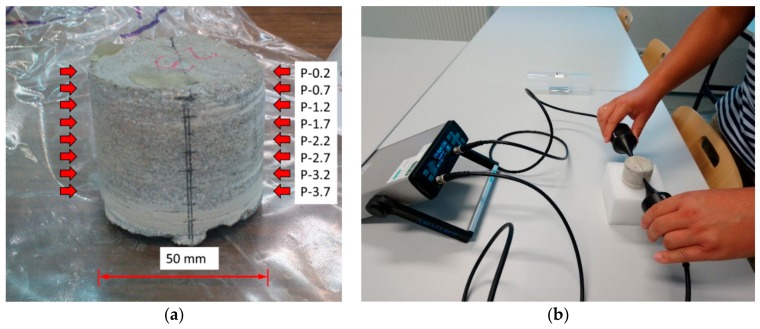
The ultrasonic method: (**a**) the arrangement of measuring points on a core sample for testing the longitudinal velocity of the ultrasonic wave *c*_L_ along the thickness of the cement mortar used to make the overlay; (**b**) the view of the core sample during the test.

From each mortar, one core sample with a diameter of 50 mm was taken in order to determine the course of the longitudinal velocity of the ultrasonic wave *c*_L_ along the thickness of the cement mortar used to make the overlay. Measuring points were marked on the lateral surfaces of these core samples at a spacing of 5 mm (Figure 3). Special ultrasound heads (Proceq AG, Schwerzenbach, Switzerland) with a frequency of 40 kHz were employed. These heads had a point contact with the test surface and are described in detail in [66]. Classical ultrasonic heads do not allow one to observe properly, the course of the longitudinal velocity of the ultrasonic wave *c*_L_ along the thickness of the cement mortar [67,68,69,70,71]. Recently, this kind of head has been increasingly adopted to test cement-based materials [72,73].

### 2.8. Determination of the Abrasion Resistance of Cement Mortars

For each mortar, three samples measuring 71 × 71 × 71 mm^3^ were prepared in order to test their abrasion resistance according to [74]. The abrasion resistance was measured as volume loss or mass loss after 16 cycles of abrasion on Boehme (FORM+TEST Seidner&Co. GmbH, Riedlingen, Germany) wheel. The samples were fastened and loaded with a force of 294 ± 3 N (after each cycle the sample was turned at 90 degrees).

### 2.9. Determination of the Subsurface Tensile Strength and Subsurface Hardness of Cement Mortars

According to [65], the subsurface tensile strength *f*_h_ of the cement mortar used to make the overlay was determined using the pull-off method on the surface of the overlay. According to [75], the subsurface hardness was determined using the sclerometric method. For subsurface hardness testing, the Schmidt hammer type N (Proceq AG, Schwerzenbach, Switzerland) was used. In each measuring point at least 9 measurements were taken.

### 2.10. Determination of the Microstructure of the Samples Using a Scanning Electron Microscope (SEM)

Then, from each mortar, 1 cubic sample of size 11 × 11 × 11 mm^3^ was taken from the subsurface zone of the cement mortar used to make the overlay. Consequently, 1 cubic sample with the same dimensions was prepared from the interphase zone between the cement mortar used to make the overlay and the concrete substrate. These samples were used for microstructural tests using a scanning electron microscope (SEM, JEOL, Tokyo, Japan). The procedure of obtaining samples was as follows: first, the drill core with a diameter of 50 mm was created in samples (Figure 1). Then, cubic samples were cut using table diamond saw. In order to analyze the microstructure of mortars, the JEOL SEM model JSM-6610A (JEOL, Tokyo, Japan) was used. It was equipped with a tungsten cathode (Tungsten Hairpin Filament). A material contrast mode of the backscattered electron (BSE, JEOL, Tokyo, Japan) detector was applied. The BSE detector had an accelerating voltage equal to 20 kV and a beam current of 40 nA at the working distance of 10 mm. The method of segmentation of pores with cement paste is based on analysis of area segmented on BSE images at different threshold levels [76]. As the threshold level increases, the area inside the pores increases, followed by pixels near the pore boundary. When the threshold level reaches a critical level, there is a significant increase in volume segmented in the BSE image around the pores. This critical level can be assumed as a threshold level for pores. It can be determined on the cumulative grayscale histogram near the inflection of the cumulative curve as the intersection point between two straight lines.

## 3. Results and Analysis

### 3.1. The Particle Size Distribution of Nanopowder Using Transmission Electron Microscopy (TEM)

**Figure 4:**
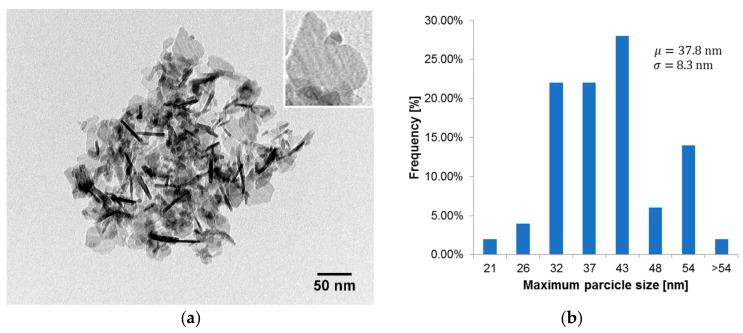
View of: (**a**) the transmission electron microscope (TEM) image of Al_2_O_3_ nanopowder particles; (**b**) the particle size distribution of Al_2_O_3_ nanopowder.

Figure 4 presents an image of the Al_2_O_3_ nanopowder particles, which was made using a TEM and the particle size distribution of Al_2_O_3_ nanopowder. Nanopowder containing 99.8% of Al_2_O_3_ with a mean particle size below 50 nm was used. The particle size distribution is based on 50 randomly chosen particles. The longest diagonal was assumed as particle size.

### 3.2. The Consistency and Bulk Density of Fresh Mortars

**Figure 5:**
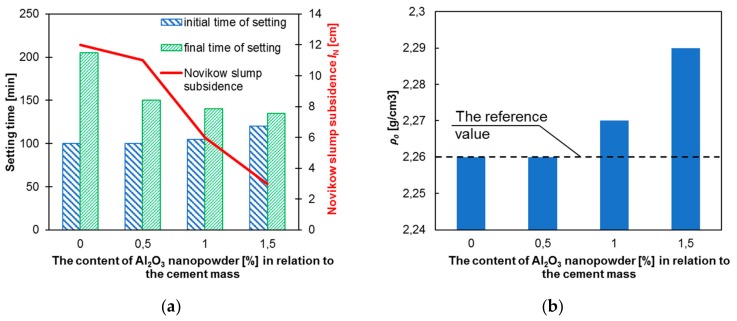
Test results of fresh cement mortars: (**a**) Novikow slump test and setting times; (**b**) bulk density.

Figure 5 presents the dependence of Novikow slump test and setting times (Figure 5a), and the bulk density (Figure 5b) of fresh cement mortars on the content of Al_2_O_3_ nanopowder.

Figure 5a presents that the final setting time is shortened (maximum by about 34%) and the initial time is slightly longer (maximum by about 20%) together with the increase of content of Al_2_O_3_ nanopowder in the mortar. The probable reason the decrease of final setting time is faster hydration [76]. It should also be noted that as the content of Al_2_O_3_ nanopowder in the mortar increases, its consistency changes quickly (except for 0.5%). For the reference mortar, the Novikow cone dropped to 12 cm. For the addition of 1% of Al_2_O_3_ nanopowder, it was 6 cm and for 1.5% it was 3 cm. It can be seen from Figure 4b that the density of the fresh mortar increases with an increasing content of Al_2_O_3_ nanopowder in its composition. However, this increase is not greater than 1.5%.

### 3.3. The Mechanical Properties and Porosity of Hardened Cement Mortars

**Figure 6:**
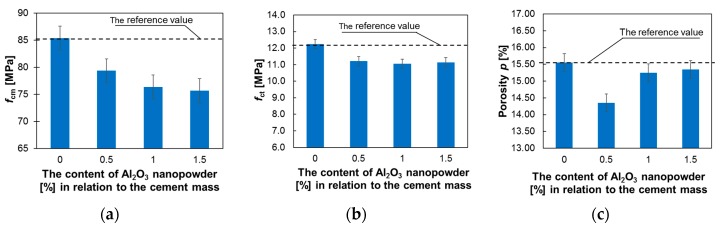
Test results of mechanical properties and porosity of hardened mortars: (**a**) compressive strength, (**b**) flexural strength and (**c**) porosity.

Figure 6 presents the results of testing the compressive strength, flexural strength and porosity of mortars differing in terms of their percentages of Al_2_O_3_ nanopowder.

Figure 5a presents that the compressive strength of all the tested mortars decreases with increasing amounts of Al_2_O_3_ nanopowder in their composition. For the addition of 0.5% Al_2_O_3_ nanopowder, this decrease became about 7%; for 1% it was about 10.6%; and for 1.5% it was about 11.4% compared to the reference mortar. In the literature there are papers which report that generally the addition of Al_2_O_3_ nanopowder can increase the compressive strength of mortars [77,78,79]; however, not at all cases. For example, some results presented, for example, those in papers [80,81,82], say that although the compressive strength of mortar with addition of Al_2_O_3_ nanopowder increased after three and seven curing days, the compressive strength after 28 days was lower than the value of reference mortar. The flexural strength was also reduced by approximately 10%, regardless of the nanopowder content (Figure 5b). For the addition of 1% and 1.5% nanopowder, the porosity is reduced to a maximum of about 2% in comparison with the reference mortar, and for the addition of 0.5% of Al_2_O_3_ nanopowder, the decrease of the porosity is about 7.7%. The possible reason for the decrease in mechanical properties could be related to low water/binder ratio of examined mortars (0.3). The addition of Al_2_O_3_ nanopowder decreases water/cement ratio and it can affect development of hydration.

### 3.4. The Pull-Off Adhesion of the Cement Mortar Used to Make the Overlay to the Concrete Substrate

Table 2 presents the test results of the pull-off adhesion *f*_b_ of the cement mortar used to make the overlay to the concrete substrate. The results presented in Table 2 confirm the known fact that the application of a bonding agent prior to the application of the cement mortar used to make the overlay increases the pull-off adhesion *f*_b_. However, in this case, the mechanical treatment of the concrete substrate surface has a much greater impact on the increase of this adhesion. This is especially noticeable for the shot-blasted surface (increase by approximately 67% compared to the raw surface R). Such a great increase of adhesion presents how important the way substrate treatments and the morphology of their surface are handled before laying the cement mortar used to make the overlay. In paper [17] they referred that in the case of shot-blasted surface, the reason for the increase of adhesion is in increase of the effective surface area and the surface exposure of the coarse aggregate. For the shot-blasted surface S, the largest increase in pull-off adhesion *f*_b_ was noted for the mortar with the addition of 0.5% of Al_2_O_3_ nanopowder. Table 2 also presents that the values of the coefficients of variation have a maximum value of about 7.13% for the raw, shot-blasted and shot-blasted surface with a bonding agent. On the other hand, for the raw surface with bonding agent, these values are several times higher (about 19% for the reference mortar and a maximum of about 24% in the mortars with the addition of Al_2_O_3_ nanopowder).

**Figure 7:**
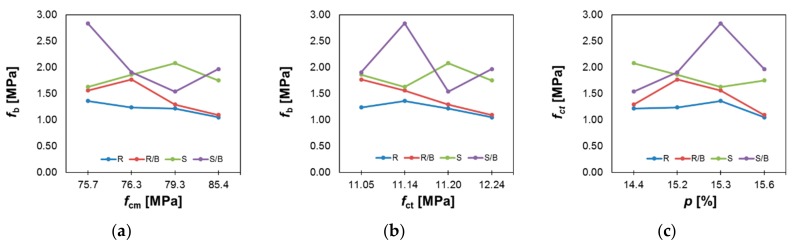
Relationship between the pull-off adhesion *f*_b_ values and the values of: (**a**) compressive strength *f*_cm_, (**b**) flexural strength *f*_ct_ and (**c**) porosity *p.*

Diversely, Figure 7 presents the relationship between the pull-off adhesion *f*_b_ values and the compressive strength *f*_cm_ (Figure 7a), flexural strength *f*_ct_ (Figure 7b) and porosity *p* (Figure 7c) for the mortars.

Figure 7 presents that for the raw surface, and the raw surface with the bonding agent; the value of *f*_b_ generally decreases as the compressive and flexural strength of the cement mortar used to make the overlay decreases. In this case, there is no clear relationship between porosity and adhesion. It can also be seen that the application of the bonding agent before applying the cement mortar used to make the overlay increases the *f*_b_ value. For the shot-blasted surface and shot-blasted surface with a bonding agent, the *f*_b_ values are higher than for the raw surfaces. In this case, there is no clear relationship between the values of *f*_b_ and compressive strength, flexural strength and porosity. However, when considering only the mortars with the addition of Al_2_O_3_ nanopowder, it can be seen that with an increasing compressive strength, the *f*_b_ decreases for the shot-blasted surface and for the shot-blasted surface with the bonding agent. It is also visible, that the *f*_b_ values decrease with an increasing porosity for the shot-blasted surface and increase for the shot-blasted surface with the bonding agent.

### 3.5. The Course of the Longitudinal Velocity of the Ultrasonic Wave along the Thickness of the Cement Mortar Used to Make the Overlay

**Figure 8:**
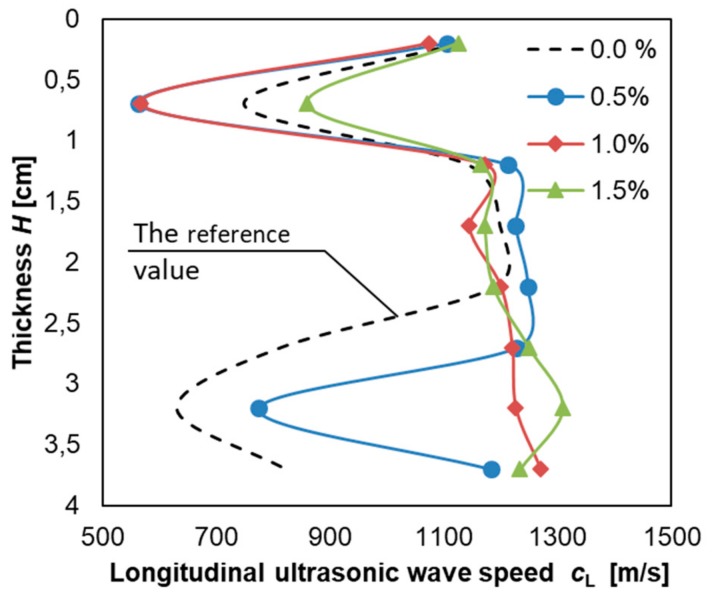
The results of the course of the longitudinal velocity of the ultrasonic wave *c*_L_ along the thickness *H* of the cement mortar used to make the overlay.

Figure 8 presents the course of the longitudinal velocity of the ultrasonic wave *c*_L_ along the thickness *H* of the cement mortar used to make the overlay.

It can be concluded from Figure 8, that the values of the longitudinal velocity of the ultrasonic wave *c*_L_ for the mortars with the addition of Al_2_O_3_ nanopowder (except mortar with addition of 0.5%) differ considerably from the values obtained for the mortar without Al_2_O_3_ nanopowder. That is especially visible in the lower section in Figure 8 (increase of the longitudinal velocity of the ultrasonic wave *c*_L_ value by a maximum of about 200% for the mortars with a 1% and 1.5% addition). For the mortar with the addition of 0.5%, the course of ultrasonic velocity is similar but the increase by about 23% can still be seen. This is especially evident at a thickness of between 3 and 4 cm. In Figure 8, there are two peaks (in upper zone and bottom zone) which can be caused by patch grabbing the surface of the cement mortar used to make the overlay (the upper zone) and the wall effect [83]. These results are similar to those obtained by Stawiski [84,85], who presented that the quality of the cement mortar in the top zone of an overlay can be much worse than the quality of the cement mortar in the middle and bottom zones. Such great differences in the speed of ultrasonic wave may indicate an increase in the homogeneity of the mortar in the zone close to the interphase zone. Bearing the above in mind, in order to analyze the material microstructure in the interphase zone, samples of the mortar with the addition of 0.5% of Al_2_O_3_ nanopowder, which was laid on a concrete substrate prepared by shot-blasting, were taken.

### 3.6. The Abrasion Resistance of Cement Mortars

**Figure 9:**
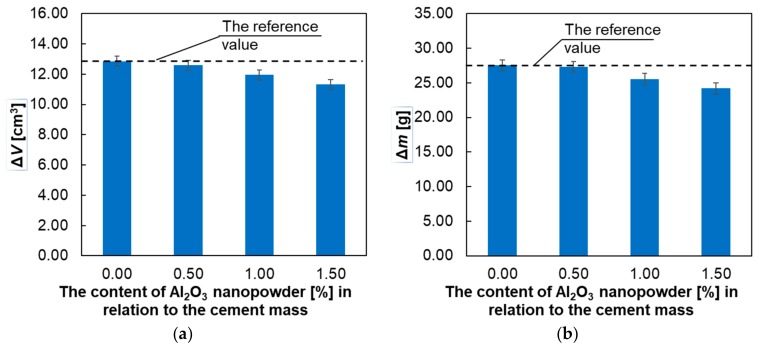
Test results of the abrasion resistance of the hardened mortars: (**a**) volume loss *ΔV*; (**b**) mass loss *Δm.*

Figure 9 presents the relationship between the abrasion resistance of the mortars tested on the percentage of Al_2_O_3_ nanopowder.

Figure 9 shows that the abrasion resistance of all the mortars tested with the addition of Al_2_O_3_ nanopowder increased as the percentage of nanopowder increased. The maximum increase in abrasion resistance was observed for the addition of 1.5% Al_2_O_3_ nanopowder.

### 3.7. The Subsurface Tensile Strength and Subsurface Hardness of Cement Mortars

**Figure 10:**
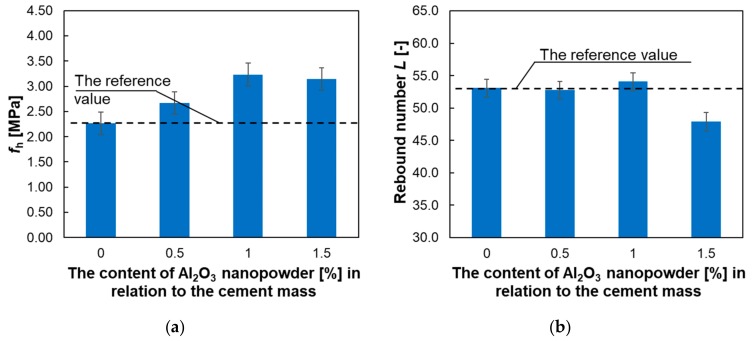
Test results of the cement mortars: (**a**) subsurface tensile strength *f*_h_; (**b**) subsurface hardness defined by the rebound number *L* obtained using the sclerometric method.

In turn, Figure 10 presents the dependence of subsurface tensile strength and the subsurface hardness of the cement mortar used to make the overlay, on the percentage of Al_2_O_3_ nanopowder.

Figure 10 shows that the addition of Al_2_O_3_ nanopowder increases the subsurface tensile strength of the cement mortar used to make the overlay. The maximum increase was about 61% and was observed for the addition of 1% of Al_2_O_3_ nanopowder. For the 0.5% of Al_2_O_3_ nanopowder, this increase was about 17%, and for the 1.5% of Al_2_O_3_ nanopowder it was about 42%. In turn, the hardness of the tested mortars assessed using the sclerometric method does not change significantly, except for the mortar with the addition of 1.5% of Al_2_O_3_ nanopowder, where the hardness decreased by about 10%.

### 3.8. The Microstructure of the Samples Using a Scanning Electron Microscope (SEM)

**Figure 11:**
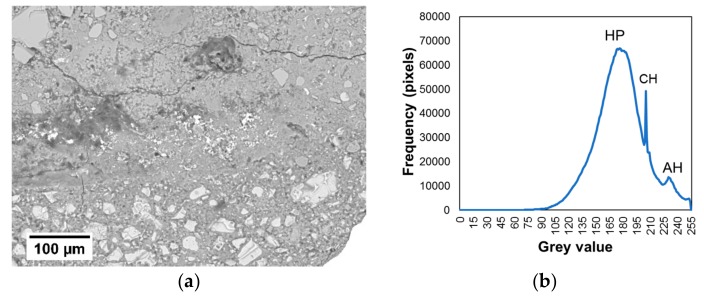

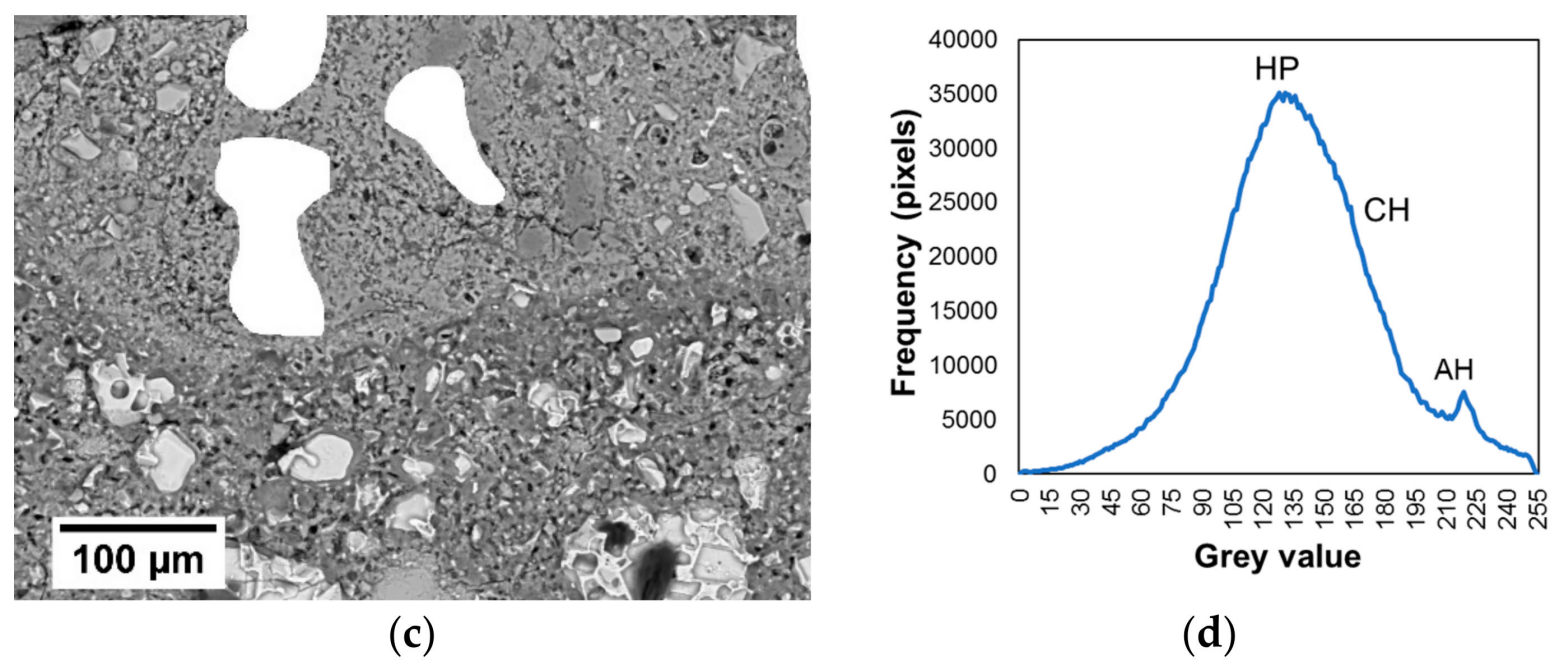
Exemplary backscattered electron (BSE) images of the samples cut from the interphase zone of the cement mortar used to make the overlay with the concrete substrate (**a**,**c**), which were made using a scanning electron microscope (SEM). Gray scale histograms (**b**,**d**). Figures (**a**,**b**) refer to the reference mortar without Al_2_O_3_ nanopowder and (**c**,**d**) refer to the mortar modified with 0.5% of Al_2_O_3_ nanopowder.

**Figure 12:**
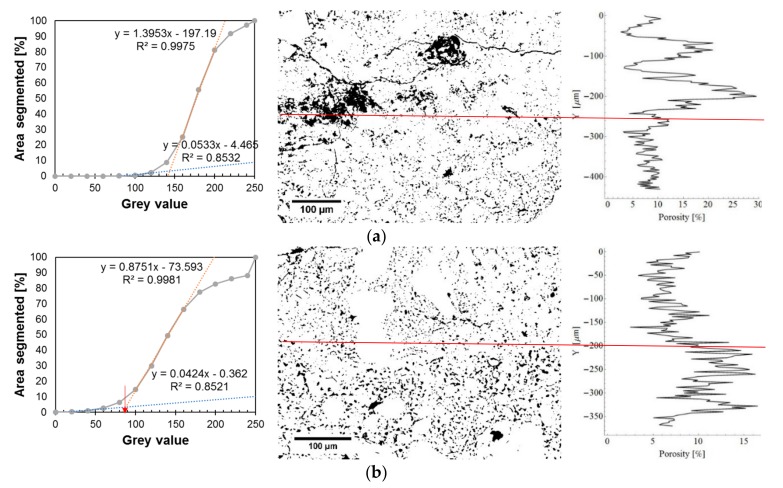
Exemplary cumulative gray scale histograms, images of segmented pores and charts of the fractional share of pores along the thickness of the interphase zone for the mortar: (**a**) without Al_2_O_3_ nanopowder; (**b**) modified with the addition of 0.5% of Al_2_O_3_ nanopowder.

The results of tests using a SEM on the samples taken from the interphase zone are presented below. Gray scale histograms and the BSE images of the samples cut from the interphase zone of the cement mortar used to make the overlay with the concrete substrate made using a SEM are presented in Figure 11. Figure 11a refers to the reference mortar, and Figure 12b applies to the mortar with the addition of 0.5% of Al_2_O_3_ nanopowder. The analyzed areas of the 0.588 × 0.433 mm^2^ interphase zone are presented on the left. To analyze the cement matrix itself, the aggregate was cut out from both these images. This procedure was previously used in, e.g., [76]. The histograms presented in Figure 11 present three visible peaks showing pores, hydration products (HP) and anhydrous cement grains (AH), and one smaller peak which corresponds to the calcium hydroxide (CH).

Alternatively, Figure 12 presents the cumulative gray scale histograms, images of segmented pores and charts of the fractional share of pores along the thickness. Figure 12a refers to the reference mortar, and Figure 12b applies to the mortar with the addition of 0.5% of Al_2_O_3_ nanopowder. The gray scale charts indicate the pore gray scale thresholds with a red arrow (following the procedure described in [76]). These values were used to generate images of the segmented pores, which are presented in the middle of the figure. On the right side of the figure, diagrams displaying the fractional share of pores along the thickness are presented. The red line indicates the center of the interphase.

Figure 11 and Figure 12 show that the mortar with the addition of 0.5% of Al_2_O_3_ nanopowder has a lower fractional share of pores compared to the reference mortar. The average value of the fractional share of pores for the reference mortar is about 10.18%, and for the mortar with the addition of nanopowder it is 8.38%. The use of 0.5% of Al_2_O_3_ nanopowder results in a reduction of the fractional share of pores in the interphase zone by about 18% compared to the reference mortar.

**Figure 13:**
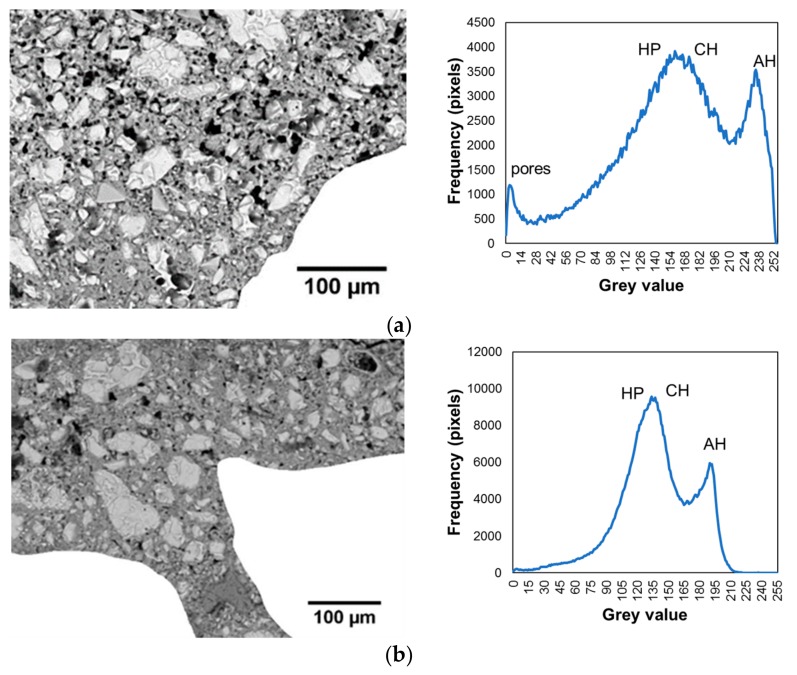
Exemplary backscattered electron (BSE) images of the samples taken from the subsurface zone of the cement mortar used to make the overlay with the concrete substrate, which were made using scanning electron microscope (SEM); and gray scale histograms for the mortar: (**a**) without Al_2_O_3_ nanopowder; (**b**) modified with the addition of 0.5% Al_2_O_3_ nanopowder.

The results of tests using SEM on the samples taken from the subsurface zone of the cement mortar used to make the overlay are presented below. Figure 13 presents electron microscope (BSE) images and gray scale histograms.

The gray scale histogram for the reference mortar (Figure 13a) presents three large peaks and one much smaller peak. Large peaks are related to the pores, hydration products (HP) and anhydrous cement grains (AH), and a much smaller peak is related to the calcium hydroxide (CH). In turn, on the histogram presented in Figure 13b, the peak related to the pores is not clearly visible for the mortar with 0.5% of Al_2_O_3_ nanopowder. Figure 13 shows that the subsurface zone of the mortar with 0.5% of Al_2_O_3_ nanopowder is less porous compared to the reference mortar.

**Figure 14:**
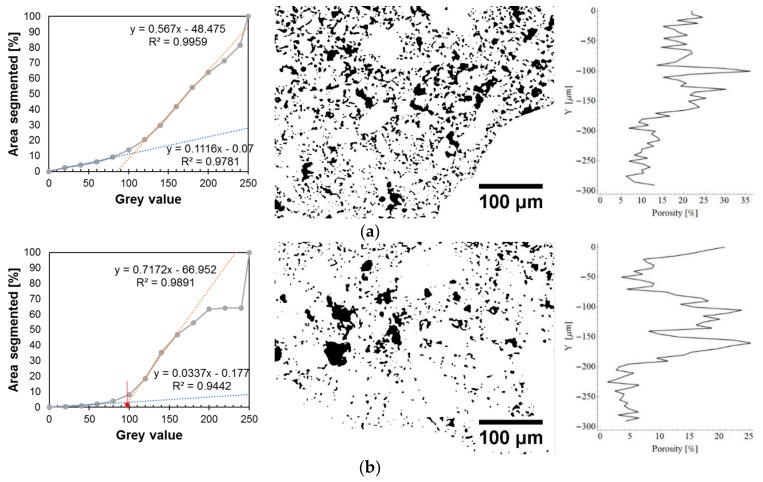
Exemplary cumulative gray scale histograms, images of segmented pores and charts of the fractional share of pores along the thickness within the subsurface zone for the mortar: (**a**) without Al_2_O_3_ nanopowder; (**b**) modified with the addition of 0.5% of Al_2_O_3_ nanopowder.

As a result of binarization, Figure 14 presents the cumulative gray scale histograms, images of segmented pores and charts of the fractional share of pores extracted from the BSE images presented in Figure 13. Figure 14 refers to the subsurface zone of the reference mortar used to make the overlay (Figure 14a) and for the mortar with 0.5% of Al_2_O_3_ nanopowder (Figure 14b). On the cumulative gray scale histograms, the pore gray scale threshold is marked with a red arrow (following the procedure presented in [76]). Then, the fractional fraction of pores was determined for the reference mortar (18.54%) and for the mortar with the addition of 0.5% of Al_2_O_3_ nanopowder (11.16%).

Figure 14 shows that the mortar with 0.5% of Al_2_O_3_ nanopowder has a smaller fractional share of pores at a thickness of 200 µm from the surface of the cement mortar used to make the overlay. The value of this fractional share of pores is about 15%, and for the reference mortar the value is about 20%. At a depth of less than 200 µm, the value of the fractional share of pores for both mortars decreases and is about 5% for the mortar with the addition of 0.5% of Al_2_O_3_ nanopowder and about 10% for the reference mortar.

**Figure 15:**
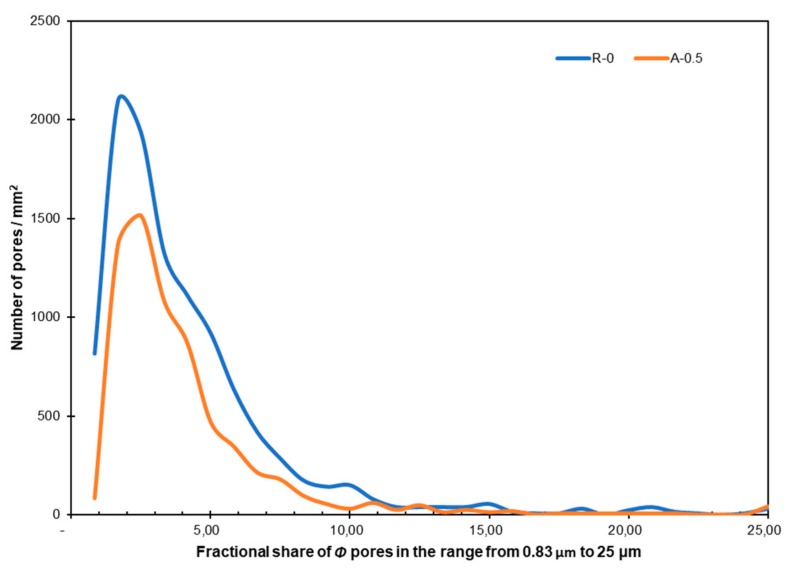
The fractional share of pores in the range of 0.83–25μm/mm^2^ in the reference mortar and in the mortar with 0.5% of Al_2_O_3_ nanopowder.

Figure 15 presents the fractional share of pores in the range of 0.83–25 μm/mm^2^ in the reference mortar and in the mortar with 0.5% of Al_2_O_3_ nanopowder.

Figure 15 shows that the matrix with 0.5% of Al_2_O_3_ nanopowder is less porous compared to the reference mortar. That is visible, especially for pores with sizes in the range of 0.83–10 µm. In both analyzed matrices, the largest number of pores was in the range of 2 to 3 µm (about 2100 pores per 1 mm^2^ in the reference mortar and about 1500 pores per 1 mm^2^ in the case of the mortar with the addition of 0.5% of Al_2_O_3_ nanopowder). The total fractional share of pores in the matrix was about 18.5% for the reference mortar and about 11.2% for the mortar with the addition of 0.5% of Al_2_O_3_ nanopowder.

## 4. Conclusions

The article focuses on the effect of applying aluminum oxide (Al_2_O_3_) nanopowder to cement mortar used to make the overlay, on the adhesion of this overlay to concrete substrate, and also its effect on the functional properties of the cement mortar used to make the overlay. The following conclusions cane be drawn:

1. The studies conducted of setting times, tests using the Novikow cone and tests of the bulk density of the fresh cement mortars showed that, with an increasing content of Al_2_O_3_ nanopowder in the cement mortar, the consistency of the mixture deteriorates. The exception is the addition of 0.5% of Al_2_O_3_ nanopowder, for which the consistency of the mix is at a lower level when compared to the mortar without nanopowder in its composition. In addition, the increase in content of Al_2_O_3_ nanopowder, compared to the reference mortar, causes the shortening of the initial setting time and extending of the final setting time. It was found that the bulk density of fresh mortar is higher, together with an increase of the content of Al_2_O_3_ nanopowder. However, this was not the case for the addition of 0.5%, for which the density is at the same level in relation to the reference mortar.
2. It was found that the mechanical properties and porosity of the hardened mortar do not depend on the addition of Al_2_O_3_ nanopowder. Only the addition of 0.5% of Al_2_O_3_ nanopowder decreases the porosity in comparison to the reference mortar without nanopowder in its composition. In turn, the addition of 0.5%, 1.0% and 1.5% of Al_2_O_3_ nanopowder results in a lower compressive and flexural strengths than for the reference mortar.
3. The results obtained using the pull-off method show that the addition of 0.5% of Al_2_O_3_ nanopowder has a positive effect on the pull-off adhesion *f*_b_ of the cement mortar used to make the overlay to the concrete substrate. It was shown that the addition of Al_2_O_3_ nanopowder considerably reduces the coefficient of variation and standard deviation of the values obtained of the pull-off adhesion *f*_b_. This was confirmed by ultrasonic tests, which presented that the addition of Al_2_O_3_ nanopowder in cement mortar has a very positive effect on the longitudinal wave speed *c*_L_ at a distance of about 15 mm from the interphase between the cement mortar used to make the overlay and the concrete substrate. This was also confirmed by the research carried out using a scanning electron microscope (SEM), which proved that the reason for improving the adhesion is the fact that the mortar with 0.5% of Al_2_O_3_ nanopowder is less porous compared to the reference mortar.
4. The results of the abrasion resistance tests were that when using 1.0% and 1.5% of Al_2_O_3_ nanopowder, the abrasion resistance of the cement mortar used to make the overlay increased in comparison with the reference mortar. It was also found that the mortar made with 0.5%, 1.0% and 1.5% of Al_2_O_3_ nanopowder had a lower subsurface tensile strength in relation to the reference mortar. On the other hand, the subsurface hardness of the cement mortar used to make the overlay, detected using the sclerometric method, does not depend on the addition of Al_2_O_3_ nanopowder. The most favorable results, in terms of lower abrasion resistance and higher subsurface tensile strength, are mainly brought about by the use of 0.5% of Al_2_O_3_ nanopowder. The studies performed using SEM confirmed that the reason for the improvement in abrasion resistance and subsurface tensile strength is the fact that the mortar with the addition of 0.5% of Al_2_O_3_ nanopowder is less porous than the reference mortar.

## Figures and Tables

**Table 1:** Mix designs of cement mortars used to make the overlays with the addition of aluminum oxide (Al_2_O_3_) nanopowder (per 100 g of sand).

Content of Al_2_O_3_ Nanopowder	Al_2_O_3_ Nanopowder	Cement type CEM I 42.5 R	Fine Aggregate (Sand)	Polycarboxylate-Based Superplasticizer	Water
(% of the mass of cement)	(g)				
0	0	73.30	100.00	0.37	22.00
0.5	0.37	73.30	100.00	0.37	22.00
1.0	0.73	73.30	100.00	0.37	22.00
1.5	1.10	73.30	100.00	0.37	22.00

**Table 2:** Test results of the pull-off adhesion *f*_b_ of the cement mortar used to make the overlay to the concrete substrate.

Surface	Content of Al_2_O_3_ Nanopowder	Mean Values	Standard Deviation	Coefficients of Variation
	(% of the Mass of Cement)	(MPa)	(-)	(%)
R (Patch grabbed surface)	0	1.05	0.07	6.67%
	0.5	1.22	0.04	3.07%
	1.0	1.24	0.04	3.29%
	1.5	1.36	0.03	2.42%
R/B (patch grabbed with bonding agent)	0	1.10	0.21	19.09%
	0.5	1.29	0.30	22.87%
	1.0	1.77	0.42	23.54%
	1.5	1.56	0.30	19.38%
S (shot-blasted surface)	0	1.75	0.05	2.65%
	0.5	2.08	0.08	4.08%
	1.0	1.86	0.05	2.85%
	1.5	1.63	0.06	3.68%
S/B (shot-blasted surface with bonding agent)	0	1.97	0.09	4.62%
	0.5	1.54	0.11	7.13%
	1.0	1.91	0.11	5.65%
	1.5	2.84	0.06	2.16%
